# Integrative Biomarker Assessment of the Influence of Saxitoxin on Marine Bivalves: A Comparative Study of the Two Bivalve Species Oysters, *Crassostrea gigas*, and Scallops, *Chlamys farreri*

**DOI:** 10.3389/fphys.2018.01173

**Published:** 2018-08-21

**Authors:** Ruiwen Cao, Dan Wang, Qianyu Wei, Qing Wang, Dinglong Yang, Hui Liu, Zhijun Dong, Xiaoli Zhang, Qianqian Zhang, Jianmin Zhao

**Affiliations:** ^1^Muping Coastal Environmental Research Station, Yantai Institute of Coastal Zone Research, Chinese Academy of Sciences, Yantai, China; ^2^Key Laboratory of Coastal Biology and Biological Resources Utilization, Yantai Institute of Coastal Zone Research, Chinese Academy of Sciences, Yantai, China; ^3^University of Chinese Academy of Sciences, Beijing, China

**Keywords:** saxitoxin, *Crassostrea gigas*, *Chlamys farreri*, oxidative stress, immunotoxicity

## Abstract

Harmful algae blooms have expanded greatly in recent decades, and their secreted toxins pose a severe threat to human health and marine ecosystems. Saxitoxin (STX) is a main paralytic shellfish poison naturally produced by marine microalgae of the genus *Alexandrium*. Despite numerous studies have assessed the impacts of STX on marine bivalves, comparative *in vivo* study on the toxicity of STX on bivalves with distinct accumulation ability (such as oysters and scallops) has been seldom investigated. The aim of this study was to identify whether distinct sensitivity exists between oysters, *Crassostrea gigas*, and scallops, *Chlamys farreri* under the same amount of STX exposure using multiple biomarker responses. The responses of different biochemical markers including oxidative stress markers (catalase, superoxide dismutase, glutathione *S*-transferase, and lipid peroxidation) and immunotoxicity biomarkers (hemocyte phagocytosis rate, reactive oxidative species production, and DNA damages) were evaluated in bivalves after 12, 48, and 96 h of exposure to STX. The integrated biomarker responses value combined with two-way ANOVA analysis suggested that STX posed slightly severer stress on scallops than oysters for the extended period of time. This study provided preliminary results on the usefulness of a multi-biomarker approach to assess the toxicity associated with STX exposure in marine bivalves.

## Introduction

In recent decades, harmful algae blooms (HABs) have increased in frequency and expanded in spatial extent worldwide, which represents a risk for marine ecosystems due to the excreted toxins of HABs (Bricelj and Shumway, 1998; Lagos, 1998; Wang and Wu, 2009; Klemas, 2011; Rodrigues et al., 2012). Marine bivalves constitute a major taxonomic group in estuarine and coastal regions, and play important roles in community structure and ecosystem functioning (Dumbauld et al., 2009). In the meantime, bivalve species encounter mass mortalities while confronting HABs (Anderson et al., 2000). In recent studies, negative effects on neural function and energy metabolism, combined with behavioral functions such as feeding, valve closure, cardiac activity and respiration, have been observed in bivalve species exposed to harmful marine algae and their released toxins (Perovic et al., 2000; Moroño et al., 2001; Colin and Dam, 2003; Twiner et al., 2004; Estrada et al., 2007; Ramos and Vasconcelos, 2010; Haberkorn et al., 2011; Basti et al., 2016). In addition, some types of marine toxins can lead to reduced growth, reproduction, and survival rates of bivalve species (Blanco et al., 2006; Shumway et al., 2006; Samson et al., 2008).

Saxitoxin is a marine toxin produced in large quantities during the massive episodic proliferation of dinoflagellates of the ubiquitous and hazardous genera *Alexandrium* and *Gymnodinium* (Lagos, 1998; Rodrigues et al., 2012). STX can lead to detrimental effects in marine species and cause paralytic shellfish poisoning (PSP) in humans through trophic transfer along the food chain (Landsberg et al., 2006). STX is known as a neurotoxin that specifically targets voltage-dependent sodium channels and calcium and potassium channels, resulting in neuromuscular paralysis and metabolic stress (Narahashi, 1998; Llewellyn, 2006). In addition to acting as a neurotoxin, STX can also induce severe oxidative stress in vertebrate cell lines (Melegari et al., 2012). As suspension-feeders, bivalves readily concentrate and bioaccumulate toxins by ingesting harmful algae (Bricelj and Shumway, 1998; Estrada et al., 2010). STX may induce declined reproduction and growth rates in marine bivalves, which could be a major cause of mortality in natural populations (Shumway et al., 2006).

In the meantime, STX has also shown to cause various biochemical and cellular alterations in bivalves, including antioxidant responses, immune defenses, and detoxification processes (Mello et al., 2013; Núñez-Acuña et al., 2013; Astuya et al., 2015; Detree et al., 2016; Abi-Khalil et al., 2017). Despite numerous studies have investigated the impacts of STX on marine bivalves, those studies mainly focus on few facets of the toxic effects caused by STX. Due to the variation in the mechanisms involved in the organisms when they are exposed to the pollutant, results interpretation from a combination of the biomarkers has more advantage than interpreting single biomarker results. As results interpretation from multi-biomarker could provide the overall impact of a specific pollutant (i.e., STX), the integrated biomarker responses (IBRs), as an indicator of environment stress, has been widely used in stress responses and ecological risk assessment of marine pollutants including toxins (Beliaeff and Burgeot, 2002; Wang et al., 2011, 2018; Pain-Devin et al., 2014; Yan et al., 2014; Martínez-Ruiz and Martínez-Jerónimo, 2015, 2017; Nogueira et al., 2015; Meng et al., 2016; Teles et al., 2016; Vieira et al., 2016; Devin et al., 2017; Duarte et al., 2017; Li et al., 2017; Luna-Acosta et al., 2017; Sanchez-García et al., 2017; Sobjak et al., 2017; Valerio-García et al., 2017). In addition, the integrated approach combining conventional toxicological bioassays with predictive biomarkers in native mussels might represent an additional tool for Control Agencies and Administrators dealing with management of human risk and economical damage caused by STX secreted by harmful algal blooms (Beliaeff and Burgeot, 2002).

Recently, differences in the sensitivity to environmental stressors, including toxic chemicals, were observed in various bivalve species (Smolowitz and Shumway, 1997; May et al., 2010; Mello et al., 2010; Ballanti et al., 2012; Ivanina and Sokolova, 2013; Carregosa et al., 2014; Götze et al., 2014; Simões et al., 2015; Velez et al., 2016; Breitwieser et al., 2017; Prasetiya et al., 2017), which may be due to the differential adaptation of organisms under certain conditions. It should also be noted that there is great variation between different shellfish species in the phycotoxins accumulation capability (Shumway, 1990). In general, oysters and mussels show rapid detoxification rates in competition with other bivalve species such as scallops (Shumway, 1995; Bricelj and Shumway, 1998). Takata et al. (2008) has found that oysters released 62% of paralytic shellfish toxin (PST) within 48 h of being held in running seawater. In contrast, scallops accumulate toxins in their tissues to a greater extent because of their low metabolic rate (Young-Lai and Aiken, 1986), and toxic retention in scallops can last up to several months from the cessation of the toxic algal bloom (Shumway, 1990). Thus, we suppose that the different accumulation ability between oysters and scallops might lead to distinct sensitivity of these two bivalve species to STX. However, to the best of our knowledge, there is to date no study investigating the different sensitivity between oysters and scallops under STX exposure through using IBRs.

Thus, multi-biomarker responses of oyster, *Crassostrea gigas*, and scallop, *Chlamys farreri*, under the same amount of STX exposure were investigated in this study. Because digestive glands serve as the main organ for toxin accumulation and detoxification (Bricelj and Shumway, 1998), the physiological and biochemical parameters investigated in this study were measured in this tissue. Specifically, the present study evaluated oxidative stress markers [catalase (CAT), superoxide dismutase (SOD), glutathione *S*-transferase (GST), and lipid peroxidation (LPO)], and immunotoxic effects [hemocyte phagocytosis rate, reactive oxidative species (ROS) production, and DNA damages] of adult oysters and scallops after exposure to STX for 12, 48, and 96 h. The mRNA expression of cytochrome P450 (CYP 450) and heat-shock protein-90 (HSP 90) transcripts were also evaluated to determine the stress and detoxification responses of bivalve species in response to STX exposure. Furthermore, the IBR index was applied as a general comparison of the potential toxicity of STX on these two bivalve species.

## Materials and Methods

### Reagent

STX (NRC CRM-STX-f) was obtained from the Institute for Marine Biosciences (National Research Centre, Halifax Regional Municipality, NS, Canada), and stored in the dark at 4°C to avoid photolysis. The stock solution was diluted to 20 μg/L in filter-sterilized seawater (FSSW).

### Experimental Conditions

Adult oysters, *C. gigas* (5–7 cm long) and scallops, *C. farreri* (4–6 cm long) were collected from an aquaculture farm located in a relatively pristine area (Yantai, Shandong, China). The oysters and scallops (soft body weight was about 10–20 g for both oysters and scallops) were acclimated for 2 weeks in aerated seawater (salinity 31.2 ± 0.5‰) at a temperature of 15.3 ± 0.2°C. The bivalves were fed daily with commercial algal blends during the acclimation period.

After acclimation, two treatment groups of each bivalve species were established (experimental STX and control group). The exposure concentration of STX (approximately 10–20 μg STX eq. 100 g^-1^ shellfish meat) used in this study is at the range of accumulated level in bivalves collected in HAB outbreak regions (0.2 to 127 × 10^3^ μg STX eq. 100 g^-1^ shellfish meat) (Bricelj and Shumway, 1998; Turner et al., 2014), which is far lower than the current EU regulation limit for human consumption of shellfish (80 μg STX eq. 100 g^-1^ shellfish meat). After the preliminary acclimation, the two bivalve species were either STX- or FSSW-injected. In the STX treatment, the oysters and scallops were directly injected with 100 μL of 20 μg/L STX. The organisms from the control group were injected with 100 μL of FSSW. The challenge experiment was carried out in 40 L aquaria with 40 organisms in each aquarium. Three replicate aquaria were used in each treatment (total of 120 individuals in each treatment), with different species separately exposed. After injection, the organisms were put back into their respective aquaria and sampled at 12, 48, and 96 h. No oyster mortality was recorded in any of the treatments during the exposure period.

At each sampling period, the digestive glands from the oysters and scallops were carefully excised and immediately frozen in liquid nitrogen and stored at -80°C for subsequent biomarker analysis. At the same time, the bivalve hemolymph samples were collected at each sampling period for the measurement of hemocyte parameters. For the measurement of oxidative stress biomarkers, a total of 12–18 individual samples (digestive glands) were dissected at each time point and pooled into six independent replicates to minimize biological variation. Also, six independent pooled samples from 12 to 18 individual samples (digestive glands) were used for real-time quantitative PCR analyses.

### Oxidative Stress Markers

The samples of the digestive glands were homogenized in phosphate buffer (50 mM potassium dihydrogen phosphate; 50 mM potassium phosphate dibasic; 1 mM EDTA; pH 7.0) and centrifuged at 10,000 *g* for 20 min at 4°C. The supernatants were used to assay the antioxidant enzymatic activities and lipid peroxidation level.

The catalase activity was determined at 240 nm based on the method described by Aebi (1984). The SOD activity was assayed by measuring its ability to inhibit the reduction of nitroblue tetrazolium (NBT), which was determined by the method described by Beauchamp and Fridovich (1971). The GST activity was determined at 340 nm according to Habig et al. (1974). The LPO level was assessed by measuring malondialdehyde (MDA) content as described by Ohkawa et al. (1979). The protein concentration was determined according to the Bradford method (Bradford, 1976), using bovine γ-globuline as a standard. All the measured parameters in this experiment were normalized for the total protein concentration of each sample.

### Hemocyte Phagocytosis and Reactive Oxygen Species Production

The hemolymph samples were withdrawn from the adductor muscle of the oysters and scallops using a 21G needle attached to a 1-ml syringe and kept on ice to avoid cell clumping. Six independent pooled samples of hemolymph were prepared using 12–18 individuals to minimize biological variation. The hemolymph sample was filtered through an 80-μm mesh sieve, centrifuged at 4°C, and then washed twice with phosphate-buffered saline (PBS) buffer (137 mM NaCl, 2.7 mM KCl, 8.1 mM Na_2_HPO_4_, and 1.5 mM KH_2_PO_4_, pH 7.4). The cells were re-suspended in 500 μL of PBS as working solution. Each pooled sample was divided into two aliquots to measure the phagocytic rate and reactive oxygen species (ROS) production.

The hemocyte phagocytosis was measured according to the method of Delaporte et al. (2003). Briefly, the hemolymph was mixed with 2.3% yellow-green FluoSpheres (YG 2.0 microns, Polyscience, Eppelheim, Germany), and then incubated at 18°C for 1 h in the dark, followed by addition of 6% formalin solution to terminate the reaction. Hemocytes were analyzed by flow cytometry using the FL-1 tunnel. The phagocytic capacities were defined as the percentage of hemocytes that engulfed three or more beads.

The determination of the intracellular ROS content was adapted from Hégaret et al. (2003). Hemocytes were incubated with 5 μL of fluorescent probe DCFH-DA (0.01 mM) at 18°C for 1 h in the dark. A FACSCalibur flow cytometer (Becton-Dickinson, San Diego, CA, United States) equipped with a 488 nm argon laser was used for functional analyses of hemocytes at the end of the incubation. The results were expressed as the geometric mean of the fluorescence [in arbitrary units (AUs)] detected in each hemolymph sample.

### Comet Assay

Six independent pooled samples of hemolymph were prepared using 12–18 individuals for the measurement of comet assay in each treatment group. A comet assay was performed following the protocol proposed by Danellakis et al. (2011) with slight modifications. Briefly, 40 μL of hemocyte suspension and 75 μL of 1.0% LMA (low melting point agar) were mixed and pipetted over a slide pre-coated with 2.0% normal melting point agarose. After the agarose was polymerized, the cover slides were removed, and a third layer of LMA was added to the slides. Then, the slides were immersed into ice-cold lysis buffer (2.5 M of NaCl, 100 mM of EDTA, 10 mM of Tris, 1% Triton X-100, 10% DMSO, pH 10.0) for 1 h. At the end of the lysis period, the slides were immersed in an alkaline electrophoresis buffer (300 mM of NaOH and 1 mM of EDTA, pH 13.0) for 20 min at 4°C, and then electrophoresis was run in the same buffer for 10 min at 25 V (300 mA, E = 0.66 V/cm). After electrophoresis, the slides were neutralized in Tris buffer (0.4 M of Tris-HCl, pH 7.5). The DNA was stained with SYBR Green I (Molecular Probe, Eugene, OR, United States) and examined with a fluorescence microscope (Olympus FV 1000, Tokyo, Japan). For each treatment group, six slides were prepared and 50 nuclei were analyzed per slide. The DNA damage was expressed as the percentage of DNA in the comet tail (% DNA in tail).

### Real-Time Quantitative PCR Analyses

The gene HSP 90 and gene CYP 450 were selected in this study for qRT-PCR assay. In addition, the gene EF-1α (AB122066.1) and gene 18S RNA (FJ588641) were used as an internal control in quantitative gene expression studies of selected genes in adult oysters and scallops, respectively. The total RNA was extracted from digestive gland tissue of the oysters and scallops using TRIzol reagent (Invitrogen, United States) following the manufacturer’s directions. The cDNA was synthesized from the DNase I-treated (Promega, United States) RNA and then mixed with 10 μL of 2× Master Mix (Applied Biosystems, United States), 4.8 μL DEPC-treated H_2_O and 0.4 μL (0.2 μM) of each forward and reverse primer to a final volume of 20 μl. Real-time quantitative PCR (qPCR) was carried out using standard protocols on an Applied Biosystems 7500 fast Real-Time PCR System (Applied Biosystems, United States). The list of primers designed for quantitative RT-PCR is shown in **Table 1**. The specificity of the qPCR products was analyzed by a dissociation curve analysis of the amplification products. The expression level of the selected genes was analyzed by using the comparative 2^-ΔΔCT^ method (Livak and Schmittgen, 2001) with target genes normalized with the selected endogenous control.

**Table 1:** Primers used for real-time quantitative PCR analysis.

	Gene name	Forward primer (5′–3′)	Reverse primer (5′–3′)	GenBank accession number
Oysters	EF1α	GAGCGTGAACGTGGTATCAC	ACAGCACAGTCAGCCTGTGA	AB122066.1
	HSP 90	GGTGAATGTTACCAAGGAAGG	GTTACGATACAGCAAGGAGATG	EF687776.1
	CYP 450 2C8	CCCTACGGTCCCTTTCCTAG	GGAGCCCGTGATCAGACTAA	XM_011451620
Scallops	18S RNA	CGTTCTTAGTTGGTGGAGCG	AACGCCACTTGTCCCTCTAA	FJ588641
	HSP 90	AACACAGTCAGTTCATCGGCTAC	TCTTCTACCTTTGGCTTGTCATC	AY362761.1
	CYP 450 family 4	TCGAGGGCGTCGTAATCC	TCTTGGTCCTGCTGAAAATGG	FJ588641


### Integrated Biomarker Response

To integrate all the measured biomarker responses into one general “stress index,” the IBR index was calculated as described by Beliaeff and Burgeot (2002) and modified by Sanchez et al. (2013). In this study, the IBR index was applied to assess the potential toxicity of STX to *C. gigas* and *C. farreri* at the three different time points (12, 48, and 96 h). The procedure for calculating the IBR index is briefly described as follows. Individual biomarker data (Xi) were compared to the reference data (X0) and log transformed to yield Yi = log Xi/X0. The general mean (μ) and standard deviation (s) of each biomarker Yi were calculated for all treatments, and Yi was standardized as Zi = (Yi-μ)/s. The biomarker deviation index (A) was calculated by using the mean of the standardized biomarker response (Zi) and the mean of the reference biomarker data (Z0) to yield Ai = Zi-Z0. To obtain IBRv2, the absolute values of A parameters calculated for each biomarker were summed to yield IBRv2 = Σ|A|. Finally, the data for each biomarker were represented in radar type graphs, and the biomarker deviation index (A) was depicted in a star plot indicating the deviation of the investigated biomarker of the STX group compared to the control group. The area above 0 reflects biomarker induction, and the area below 0 indicates biomarker inhibition.

### Statistical Analysis

All statistical analyses were performed using SPSS 13.0 statistical software (SPSS 13.0, Chicago, IL, United States). The data were expressed as the means ± standard deviation (SD). A Shapiro–Wilk test was performed to test the normality of the data. Then, the raw data were analyzed statistically by two-way analysis of variance (ANOVA). Significant differences between treatments were assessed by ANOVA combined with least significant difference (LSD) *post hoc* tests. The differences were considered statistically significant at *P* < 0.05; *P* < 0.01 was considered extremely significant.

## Results

### Oxidative Stress Markers

**FIGURE 1:**
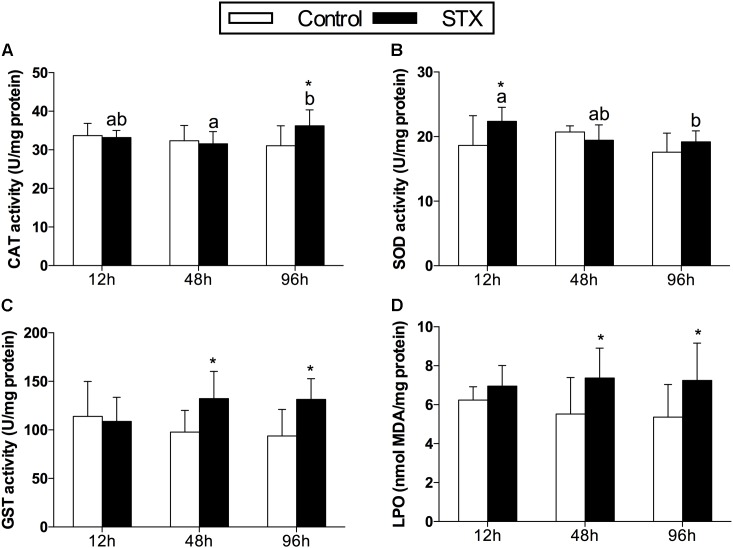
Antioxidant enzyme activities and LPO (lipid peroxidation) level in the digestive glands of *Crassostrea gigas* post-STX exposure. **(A)** CAT (catalase); **(B)** SOD (superoxide dismutase); **(C)** GST (glutathione *S*-transferase); **(D)** LPO. Each bar represents the mean ± SD (*n* = 6). Different letters indicate significant differences among treatments at the same concentration of STX exposure (*P* < 0.05). Asterisks indicate significant differences between the values for the control and STX-exposed groups (^∗^*P* < 0.05, ^∗∗^*P* < 0.01).

In oysters, the activity of CAT increased significantly (*P* < 0.05) in digestive glands under STX exposure at 96 h (**Figure 1A**). The SOD activity was one-fifth stimulated under STX exposure at 12 h compared with individuals in control group (**Figure 1B**). However, the SOD activity in STX-exposed oysters decreased significantly (*P* < 0.05) at 96 h compared to individuals at 12 h. Meanwhile, there was a significant increase (*P* < 0.05) in the GST activity and LPO level in the STX-treated group as compared to non-injected individuals at 48 and 96 h, respectively (**Figures 1C,D**).

**FIGURE 2:**
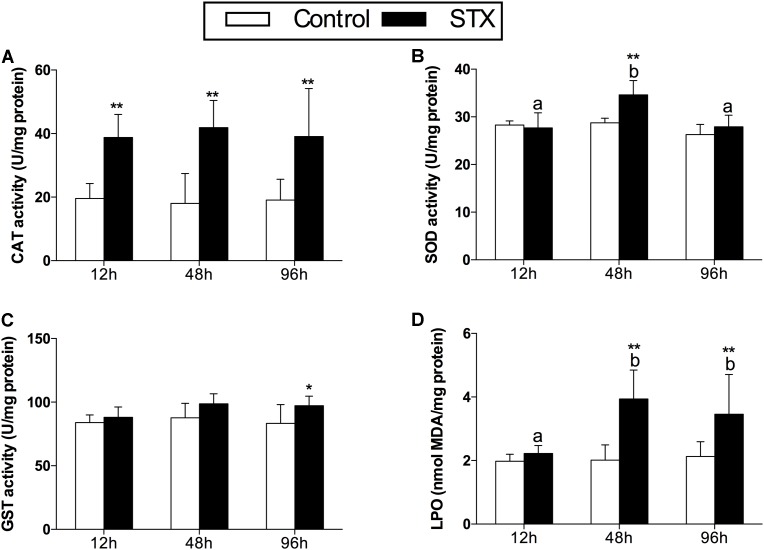
Antioxidant enzyme activities and LPO (lipid peroxidation) level in the digestive glands of *Chlamys farreri* post-STX exposure. **(A)** CAT (catalase); **(B)** SOD (superoxide dismutase); **(C)** GST (glutathione *S*-transferase); **(D)** LPO. Each bar represents the mean ± SD (*n* = 6). Different letters indicate significant differences between timepoints at the same treatment conditions (*P* < 0.05). Asterisks indicate significant differences between the values for the control and STX-exposed groups (^∗^*P* < 0.05, ^∗∗^*P* < 0.01).

In scallops, CAT activities in the STX treatment group were significantly (*P* < 0.01) increased compared to the control treatment at all three sampling time points (**Figure 2A**). Concerning the SOD activity, a significant increase (*P* < 0.01) was observed in the STX-treated scallops at 48 h compared to other treatments (**Figure 2B**). In addition, the scallop GST activity was one-sixth stimulated (*P* < 0.05) under STX exposure compared to non-exposed individuals at 96 h (**Figure 2C**). Furthermore, the LPO level increased significantly (*P* < 0.01) in the STX treatment compared to the control group at both 48 and 96 h (**Figure 2D**). Also, significant higher (*P* < 0.01) LPO level was observed in STX-exposed scallops at 48 and 96 h compared to individuals at 12 h (**Figure 2D**). Besides, significant interaction (*P* < 0.05) between STX and time was observed in SOD activity and LPO levels of scallops (**Table 2**).

**Table 2:** Two-way ANOVA: effects of STX exposure and time on the physiological parameters and gene expression in oyster *C. gigas* and scallop *C. farreri*.

			Factors/interaction		
			
			STX	Time	STX × Time
Oysters	Antioxidant enzymes activities	CAT	*F*(1,30) = 0.98	*F*(2,30) = 0.74	*F*(2,30) = 2.45
			*P* = 0.330	*P* = 0.484	*P* = 0.104
		SOD	*F*(1,30) = 2.24	*F*(2,30) = 2.08	*F*(2,30) = 2.60
			*P* = 0.145	*P* = 0.143	*P* = 0.091
		GST	***F*(1,30) = 6.14**	*F*(2,30) = 0.06	*F*(2,30) = 2.32
			***P* = 0.019**	*P* = 0.943	*P* = 0.116
		LPO	***F*(1,30) = 8.58**	*F*(2,30) = 0.11	*F*(2,30) = 0.57
			***P* = 0.006**	*P* = 0.896	*P* = 0.570
	Immune-related parameters	Phagocytosis	***F*(1,30) = 20.28**	*F*(2,30) = 1.56	*F*(2,30) = 0.76
			***P* < 0.001**	*P* = 0.231	*P* = 0.477
		ROS	*F*(1,30) = 1.13	*F*(2,30) = 0.89	*F*(2,30) = 0.53
			*P* = 0.296	*P* = 0.422	*P* = 0.593
		DNA damage	***F*(1,30) = 70.54**	***F*(2,30) = 46.34**	***F*(2,30) = 35.57**
			***P* < 0.001**	***P* < 0.001**	***P* < 0.001**
	Gene expression	HSP 90	***F*(1,30) = 11.46**	***F*(2,30) = 17.50**	***F*(2,30) = 15.48**
			***P* = 0.002**	***P* < 0.001**	***P* < 0.001**
		CYP 450	*F*(1,30) = 0.81	*F*(2,30) = 3.10	***F*(2,30) = 3.50**
			*P* = 0.375	*P* = 0.060	***P* = 0.044**
Scallops	Antioxidant enzymes activities	CAT	***F*(1,30) = 46.91**	*F*(2,30) = 0.032	*F*(2,30) = 0.22
			***P* < 0.001**	*P* = 0.968	*P* = 0.807
		SOD	***F*(1,30) = 9.20**	***F*(2,30) = 13.61**	***F*(2,30) = 6.22**
			***P* = 0.005**	***P* < 0.001**	***P* = 0.006**
		GST	***F*(1,30) = 8.87**	*F*(2,30) = 1.65	*F*(2,30) = 0.80
			***P* = 0.006**	*P* = 0.209	*P* = 0.459
		LPO	***F*(1,30) = 24.98**	***F*(2,30) = 5.24**	***F*(2,30) = 4.45**
			***P* < 0.001**	***P* = 0.011**	***P* = 0.020**
	Immune-related parameters	Phagocytosis	***F*(1,30) = 3.68**	*F*(2,30) = 2.01	***F*(2,30) = 6.28**
			***P* = 0.065**	*P* = 0.151	***P* = 0.005**
		ROS	***F*(1,30) = 17.70**	***F*(2,30) = 20.20**	***F*(2,30) = 3.58**
			***P* < 0.001**	***P* < 0.001**	***P* = 0.042**
		DNA damage	***F*(1,30) = 70.54**	***F*(2,30) = 46.34**	***F*(2,30) = 35.57**
			***P* < 0.001**	***P* < 0.001**	***P* < 0.001**
	Gene expression	HSP 90	*F*(1,30) = 3.90	*F*(2,30) = 1.06	*F*(2,30) = 0.87
			*P* = 0.058	*P* = 0.360	*P* = 0.428
		CYP 450	***F*(1,30) = 28.25**	***F*(2,30) = 15.07**	***F*(2,30) = 13.79**
			***P* < 0.001**	***P* < 0.001**	***P* < 0.001**


*Significant effects are highlighted in bold.*

### Hemocytic Parameters

**FIGURE 3:**
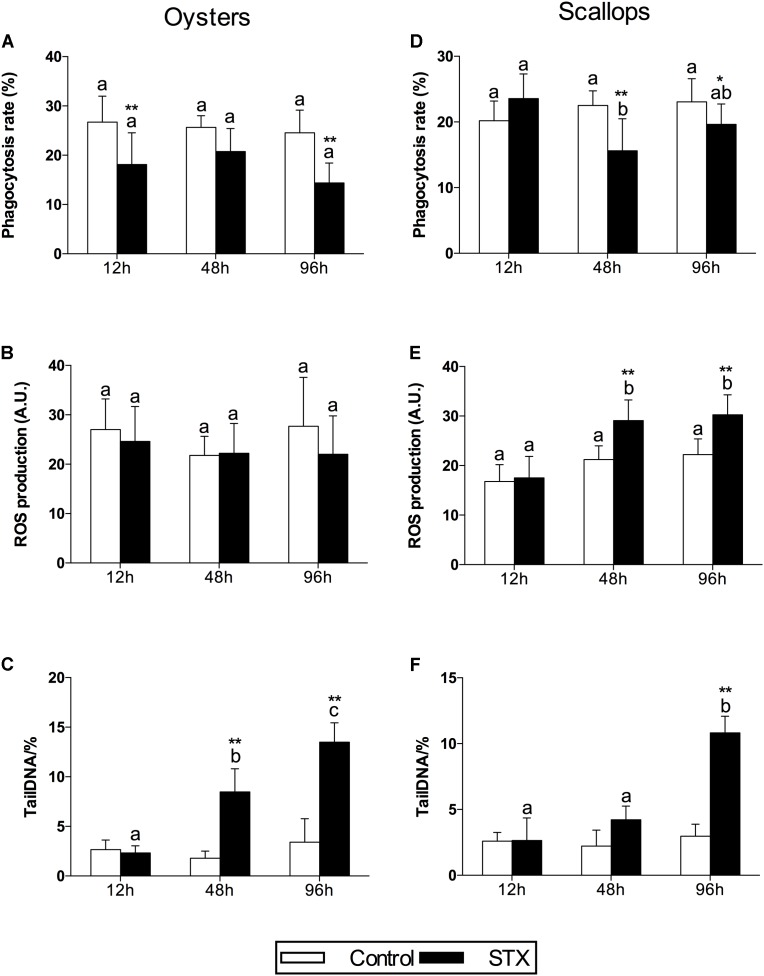
Immunotoxicity biomarkers in hemocytes of *C. gigas* **(A–C)** and *C. farreri* **(D–F)** exposed to STX for 12, 48, and 96 h. **(A,D)** Hemocyte phagocytosis rate, **(B,E)** ROS (reactive oxidative species) production, **(C,F)** DNA damage. Each bar represents the mean ± SD (*n* = 6). Different letters indicate significant differences between timepoints at the same treatment conditions (*P* < 0.05). Asterisks indicate significant differences between the values for the control and STX-exposed groups (^∗^*P* < 0.05, ^∗∗^*P* < 0.01).

In oyster hemocytes, the phagocytosis rate decreased significantly (*P* < 0.01) in the STX-treated group compared to the non-injected individuals at 12 and 96 h (**Figure 3A**). There was a slight decrease in oyster hemocyte ROS production under STX exposure at 12 and 96 h, but no significant differences were observed between exposed and non-exposed individuals (**Figure 3B**). However, the results of the comet assay showed a significant increase (*P* < 0.01) in DNA damage values in the hemocytes of oysters at 48 and 96 h (**Figure 3C**). Significant (*P* < 0.01) interaction between STX and time was observed in DNA damage of oysters (**Table 2**).

In scallops, there was a significant decrease (*P* < 0.05) in the hemocyte phagocytosis rate in the STX-treated group compared to non-exposed individuals at 48 and 96 h (**Figure 3D**). Under STX exposure, scallop hemocyte phagocytosis rate was reduced by one-third at 48 h compared to individuals at 12 h (*P* < 0.05). Moreover, an extremely significant (*P* < 0.01) increase in ROS production was observed in the STX treatment group at both 48 and 96 h (**Figure 3E**). Furthermore, the results of the comet assay revealed a one increase (*P* < 0.01) in DNA strand breaks in the scallop hemocytes under STX exposure at 96 h (**Figure 3F**). In addition, there is significant interaction (*P* < 0.05) between STX and time on all the immune parameters measured in scallop hemocytes (**Table 2**).

### Gene Expression of HSP 90 and CYP 450

**FIGURE 4:**
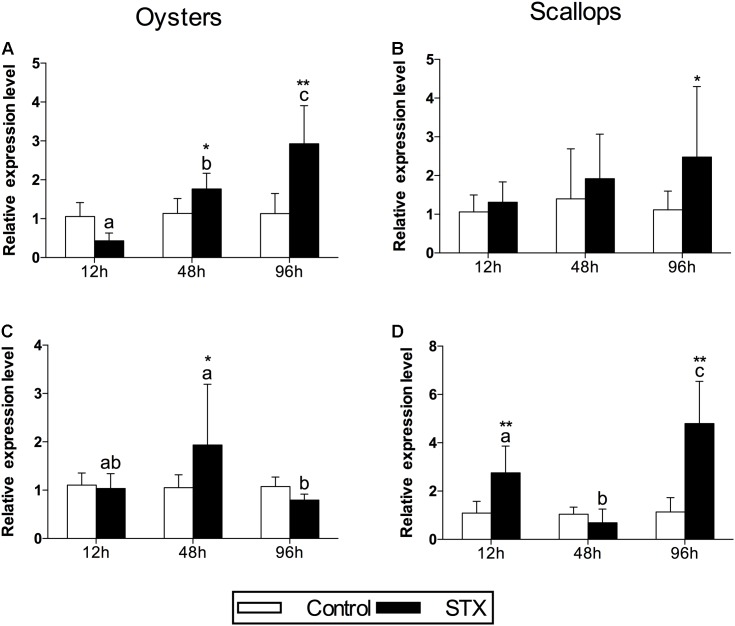
The mRNA expression profiles of the genes HSP 90 and CYP 450 in the digestive glands of oysters and scallops post-STX exposure. **(A,C)** HSP 90, **(B)** CYP 450 2C8, **(D)** CYP 450 family 4. Each bar represents the mean ± SD (*n* = 6). Different letters indicate significant differences between timepoints at the same treatment conditions (*P* < 0.05). Asterisks indicate significant differences between the values for the control and STX-exposed groups (^∗^*P* < 0.05, ^∗∗^*P* < 0.01).

In oysters, the mRNA expression of HSP 90 was significantly stimulated (*P* < 0.05) in the STX-treated group compared to non-exposed individuals at 48 and 96 h (**Figure 4A**). Besides, significant elevation in the mRNA expression of HSP 90 was observed as the exposure time increases. In STX-exposed oyster hemocytes HSP 90 transcript levels at 96 h were two-third fold higher than that at 48 h. Additionally, significantly (*P* < 0.05) stimulated expression of a CYP 450 gene (CYP 450 2C8) was observed in the oyster digestive glands after 48 h of STX exposure (**Figure 4B**). Meanwhile, there is significant interaction (*P* < 0.05) between STX and time on the mRNA expression of HSP 90 and CYP 450 2C8 in oysters (**Table 2**).

In scallops, there was a threefold increase (*P* < 0.05) in the mRNA expression of HSP 90 under STX exposure at 96 h compared to non-exposed individuals at this time point (**Figure 4C**). In addition, the mRNA expression of a CYP 450 gene (CYP 450 family 4) was increased significantly (*P* < 0.01) in the STX treatment group compared to the control group after 12 and 96 h of exposure (**Figure 4D**). In STX-exposed scallops, significant decrease (*P* < 0.05) in the mRNA expression of CYP 450 family 4 was observed at 48 h compared with individuals at 12 and 96 h. Meanwhile, mRNA expression of CYP 450 family 4 was twofold higher (*P* < 0.05) in STX-exposed scallops at 96 h than STX-exposed individuals at 12 h. Significant (*P* < 0.01) interaction between STX and time was observed in mRNA expression of CYP 450 family 4 of scallops (**Table 2**).

### Integrated Biomarker Response (IBR)

**FIGURE 5:**
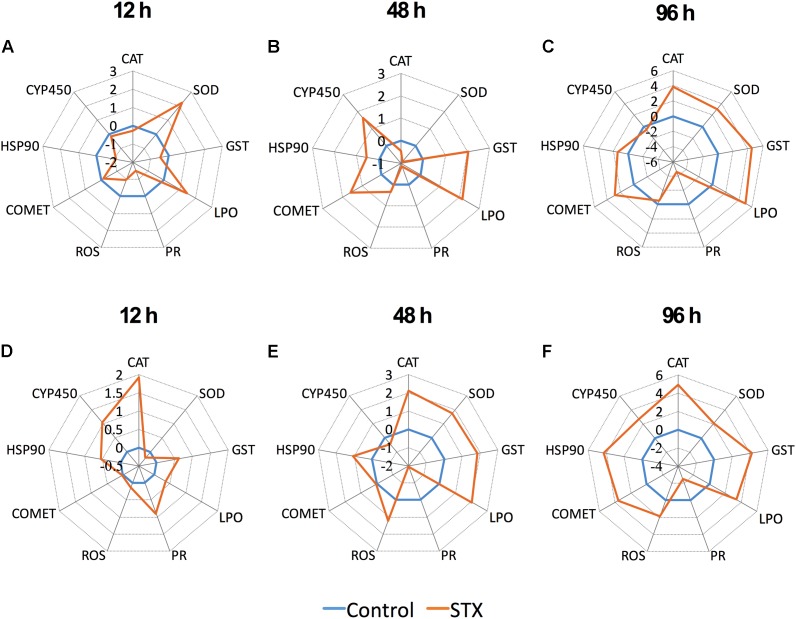
Biomarker star plots of multiple biomarker responses of *C. gigas* **(A–C)** and *C. farreri* **(D–F)** exposed to STX for 12, 48, and 96 h. CAT, catalase; SOD, superoxide dismutase; GST, glutathione *S*-transferase; LPO, lipid peroxidation; PR, hemocyte phagocytosis rate; ROS, reactive oxidative species production in hemocytes; COMET, hemocyte DNA damage; HSP 90, heat-shock protein-90; CYP 450, cytochrome P450.

**FIGURE 6:**
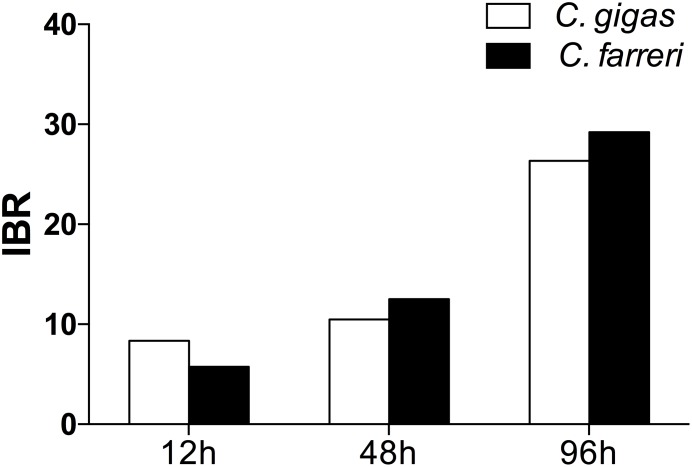
Calculated IBR index using the molecular and biochemical biomarkers measured in *C. gigas* and *C. farreri* after exposure to STX for 12, 48, and 96 h.

The transformed data of all the biomarkers at the different time points are presented as star plots in **Figure 5**. The IBR index was also calculated for each time point and showed differences in the STX-treated group in relation to the baseline (**Figure 6**). The value of the IBR index was increased with prolonged exposure to STX in the STX-treated oysters, with the highest value observed at 96 h (26.4, **Figure 6**), indicating the induction of the antioxidant enzyme responses, cellular damage, hemocyte genotoxicity, and heat-shock protein expression and the inhibition of the hemocyte phagocytosis rate and ROS production (**Figure 5C**). Similarly, the IBR index at 96 h in the STX-treated scallops was the highest among all three time points (29.2, **Figure 6**). Here, we observed stimulated antioxidant enzyme responses, cellular damage, hemocyte ROS production and genotoxicity and increased mRNA expression of HSP 90 and CYP 450 (**Figure 5F**). Additionally, the hemocyte phagocytosis rate was greatly inhibited in scallops under STX exposure (**Figure 5F**).

Meanwhile, at 48 and 96 h, the IBR index was slightly higher in scallops (10.5, 26.4) than oysters (12.5, 29.2), suggesting scallops were under severer stress than oysters in response to the same amount of STX exposure. Correspondingly, from our star graph (**Figure 5**), higher IBR value was observed in scallops than oysters at 48 h, which was associated with higher stimulation in CAT activity, SOD activity and ROS production in scallops compared with oysters. In addition, higher stimulation in ROS production and CYP 450 expression in scallops compared with oysters was observed at 96 h, which was associated with higher IBR value observed in this species than oysters at this time points.

## Discussion

Harmful algae blooms have been reported with increasing frequency worldwide due to climate change and anthropogenic activities (Heisler et al., 2008; Wang and Wu, 2009; Lapointe et al., 2015; Visser et al., 2016). Thus, the toxic mechanisms of marine biotoxins and more accurate risk evaluations associated with marine biotoxins have received increasing attention from scientific community (Accoroni et al., 2011; Munday, 2011; Trainer et al., 2014).

Recent laboratory studies have found that the toxic species associated with HABs and their secreted toxins could alter antioxidant responses and lead to cellular damage in marine organisms (Estrada et al., 2007; Amado and Monserrat, 2010; Fabioux et al., 2015; Kim et al., 2017). For example, PST-exposed clam *Ruditapes philippinarum* displayed significantly altered antioxidant enzyme activity and increased LPO levels (Choi et al., 2006). Previous *in vitro* studies on various cell lines also indicated oxidative stress induction by STX (Melegari et al., 2012). The increased oxidative stress on aquatic organisms caused by STX is expected because ROS can be produced during the process of xenobiotic detoxification (Kelly et al., 1998; Vinothini and Nagini, 2010).

Oxidative stress in organisms is mainly caused by excessive ROS production (Lushchak, 2011; Melegari et al., 2012; Zhang et al., 2013; Wang et al., 2017). High levels of ROS can be toxic to important cellular components including lipids, DNA and protein, leading to cell injury and even death in aquatic organisms (Lushchak, 2011). A wide array of low molecular weight scavengers and antioxidant enzymes function to prevent the adverse effects of ROS and maintain cellular redox homeostasis (Regoli and Giuliani, 2014). As an important antioxidant enzyme, CAT prevents the formation of excessive H_2_O_2_ through decomposing H_2_O_2_ once it is formed (Halliwell, 1974), while SOD functions as an antioxidant enzyme by catalyzing excess $O_{2}^{-}$ radicals into H_2_O_2_ and O_2_ (Halliwell, 1974). As a phase II detoxification enzyme, GST protects cells and tissues against oxidative stress by catalyzing the conjugation of the reduced form of glutathione to various xenobiotic substrates (Hayes and Strange, 1995). The above-mentioned antioxidant enzyme activities alteration has been widely used as biochemical biomarkers for oxidative stress caused by environmental stressors (Matozzo et al., 2013; Nogueira et al., 2015; Freitas et al., 2017). Based on this study, antioxidant enzymes (CAT, SOD, and GST) were generally stimulated in the digestive glands of both oysters and scallops, indicating that the antioxidant system was activated in response to the induction of oxidative stress caused by STX exposure. Correspondingly, increased CAT activity was also found in mussels, *Mytilus edulis*, exposed to the toxic cyanobacterium *Nodularia spumigena* (Kankaanpää et al., 2007). These authors suggested that the detoxification reactions could be responsible for the increased production of oxidative species. Meanwhile, the earlier stimulation of CAT activity in scallops compared with oysters might indicated higher sensitivity of scallops to STX toxicity. In addition, compared with the unchanged SOD activity in oysters, the elevated SOD activity in scallops at 48 h indicated that this taxa was under more severe oxidative stress at this time point.

However, the overwhelmed antioxidant system resulting from excessive ROS production could lead to LPO (Sevgiler et al., 2004). Elevated LPO level is a major indicator of cellular oxidative damage in organisms and is a major contributor to the loss of cell function under environmental perturbations, including toxins released by harmful algae (Moreira et al., 2016; Ricevuto et al., 2016; Valerio-García et al., 2017). Based on our results, cellular damage, as indicated by lipid peroxidation, increased significantly in both oysters and scallops after 48 and 96 h of exposure to STX. It seems that, although the antioxidant mechanisms were activated, they could not efficiently eliminate the excessive ROS, resulting in cellular damage in both bivalve species. Meanwhile, the baseline levels of LPO are threefold higher in oysters than in scallops. The differences in baseline LPO levels between the two studied species may reflect adaptations of the intertidal-dwelling oysters to higher LPO baseline levels, compared to the subtidal species such as scallops. In addition, time posed significant effects on the elevation of the scallop LPO level, which might suggest that STX pose severer oxidative damage to scallops than oysters with prolonged time exposure.

Additionally, bivalves hemocytes function as the first line of immune responses including phagocytosis, ROS production, opsonization, nodule formation and the release of immune mediators (Thomas, 1996). Inhibited phagocytosis rate and increased DNA damage of oyster and scallop hemocytes were observed in this study. Similarly, phagocytosis inhibition and genotoxicity of STX on oyster hemocytes has been observed in previous *in vitro* study (Mello et al., 2013; Abi-Khalil et al., 2017). The suppressed immune system in two bivalve species, as indicated by the inhibition of phagocytosis rate and induction of DNA damage in STX-treated oyster and scallop hemocytes, might sensitize them to future pathogen infection. In this study, STX posed no significant effect on ROS production in oyster hemocytes. Similarly, Mello et al. (2013) has found that *Alexandrium minutum* and STX could negatively affect immnunocompetence of *C. gigas* by decreasing the phagocytosis and ROS production of *C. gigas* hemocyte. However, significant stimulation of ROS production was observed in STX-exposed scallop at 48 and 96 h, which might suggest higher toxicity posed by STX on scallops than oysters with prolonged exposure. In addition, excessive ROS production in hemocytes could pose severe oxidative stress to scallops, and ROS are highly reactive molecules known to interact with sulfhydryl groups on proteins. Previous study has suggested that ROS production might be associated with the actin filament disruption in bivalve hemocytes treated with benzo(a)pyrene (Gómez-Mendikute et al., 2002). Since a bivalve’s ability to mount an efficient immune response is reliant upon the integrity and efficient functioning of hemocytes, thus, the disturbed hemocyte actin filament caused by ROS production might be partially associated with the immune suppression effect caused by STX in scallops. In addition, Abi-Khalil et al. (2017) has discovered that PSTs including STX were shown to be directly responsible for inducing apoptosis in hemocytes, a process dependent on caspase activation and independent of ROS production. Thus, we suppose that the increased DNA damage observed in oysters could be directly caused by STX toxicity rather than excessive ROS, as the ROS level in oyster hemocytes showed no change under STX exposure.

The proteins of the cytochrome P450 (CYP 450) family are known to be involved in the biotransformation of various xenobiotics in aquatic invertebrates (Snyder, 2000), and their expression was found to be disturbed in bivalve species exposed to HABs and/or their associated toxins (Mello et al., 2012, 2013; García-Lagunas et al., 2013; Huang et al., 2015). Heat shock proteins (HSPs) are molecular chaperones that assist in the refolding of stress-damaged proteins and are usually induced under stressful conditions (Moraga et al., 2005; Dong et al., 2014; Alaraby et al., 2015). The increased expression of HSP transcripts has been reported in a variety of marine organisms exposed to toxins excreted by harmful algae (da Silva et al., 2011; El Golli-Bennour and Bacha, 2011; Jiang et al., 2012; Mello et al., 2012; Núñez-Acuña et al., 2013). Correspondingly, the expression of HSP 90 and CYP 450 transcripts was upregulated in the digestive glands of oysters and scallops in the present study, indicating positive regulation by these two bivalves as part of stress responses and detoxification processes following STX exposure. However, stimulated stress responses and detoxification processes were not enough to protect the cells of the digestive glands from the toxicity caused by accumulated STX, as indicated by the increased oxidative stress and immunotoxicity in both bivalve species. The expression of HSP 90 showed similar pattern in oysters and scallops. Meanwhile, significant stimulation in the expression of a CYP 450 gene (CYP 450 family 4) were observed in STX-exposed scallops at 96 h, which was in contrast with expression of the CYP 450 gene (CYP 450 2C8) in STX-exposed oysters (showing no alteration at this timepoint). We suppose that higher STX might be accumulated in scallops than oysters at this time point, thus more CYP 450 needs to be synthesized to fulfill the complication of STX detoxification.

In this study, the IBR index was applied to compare the overall stress of STX on oysters, *C. gigas*, and scallops, *C. farreri*. This approach provides a simple tool for the visualization of biological effects by integrating different biomarker signals (Beliaeff and Burgeot, 2002). The IBR analysis showed a general stimulation of antioxidant enzyme activity, cellular damage, and immunotoxicity in the two bivalve species under STX exposure at all three investigated time points (**Figure 6**). In general, IBR index increased with extended time exposure to STX in both oysters and scallops. The highest IBR index was observed at 96 h, indicating the highest stress level at this time point. However, slightly higher IBR value was observed in STX-exposed scallops than STX-exposed oysters at both 48 and 96 h, suggesting higher stress level in scallops than oysters with prolonged exposure to STX.

Also, from our two-way ANOVA analysis results, we observed that time pose much more significant effects on scallops than oysters. Meanwhile, the significant interacted effects of STX and time on scallops were observed in most of the tested biomarkers, while only three parameters (hemocyte DNA damage, mRNA expression of HSP 90 and CYP 450) tested in oysters observed the significant interacted effects of STX and time. From both our IBR results and two-way ANOVA analysis, we conclude that STX pose severer stress on scallops than oysters as the exposure time increases. The much lower toxin detoxification ability of scallops compared with oysters, which has been investigated in previous literatures (Shumway, 1990, 1995; Bricelj and Shumway, 1998; Takata et al., 2008), might be associate with the higher sensitivity of this species than oysters in response to STX as indicated in this study. Furthermore, the different resistance capabilities between oysters and scallops could also attribute to their distinct habitats. Living in estuarine and intertidal regions, oysters are confronted with harsh and dynamic environmental stresses, including toxins excreted by harmful algae (Zhang et al., 2016). Hence, the high level of resistance and adaptive recovery of this species in response to STX acidification toxicity is expected. However, scallops live in subtidal conditions, with low levels of environmental perturbations, which could explain the high sensitivity of this species to STX toxicity, regardless of the distinct accumulation and detoxification ability between these two investigate species under STX exposure.

In the present paper, while not being lethal to oysters and scallops, STX exposure induced oxidative stress, cellular damage, and immunotoxicity indiscriminately in both oysters and scallops. Although the exposure time in this experiment is very short, the present study clearly suggested slightly higher sensitivity of scallops than oysters under exposure to the common level of STX. Thus, toxicity assessment on different bivalve species especially with distinct phycotoxin accumulation capability needs to be tested in order to better predict the ecological risk of toxins excreted by harmful algal. Meanwhile, longer time exposure to STX by oysters and scallops are required to be investigated in our future study to test whether significant difference in sensitivity to STX exists between these two bivalve species. In conclusion, the overall results of this study highlighted that the multi-biomarker analysis in bivalves might be profitably considered as an integrative tool for assessing the impact of STX, therefore should be considered in the future assessment of the environment risks of STX released during harmful algal blooms.

## Ethics Statement

All organisms used for this research were marine invertebrate bivalves and as such were not subject to Institutional Animal Care and Use Committee (IACUC; China) oversight. However, care was taken in designing experiments to limit and reduce the number animals sacrificed during the course of research. All organisms prior to and after experimentation were adequately fed, maintained at optimal densities, and routinely cared for.

## Author Contributions

RC, DW, QyW, QW, DY, HL, ZD, XZ, and QZ performed the experiments. RC, QZ, and JZ conceived and designed the experimental plan, analyzed the data, and drafted the manuscript.

## Conflict of Interest Statement

The authors declare that the research was conducted in the absence of any commercial or financial relationships that could be construed as a potential conflict of interest.
